# KDM1A epigenetically enhances RAD51 expression to suppress the STING-associated anti-tumor immunity in esophageal squamous cell carcinoma

**DOI:** 10.1038/s41419-024-07275-4

**Published:** 2024-12-06

**Authors:** Qingyuan Yang, Shiyin Wei, Cen Qiu, Chenjie Han, Zunguo Du, Ning Wu

**Affiliations:** 1 Department of Pathology, Shanghai Ninth People’s Hospital, Shanghai Jiao Tong University School of Medicine, Shanghai, China; 2 Affiliated Hospital of Youjiang Medical University for Nationalities, Baise City, Guangxi China; 3 Department of Pathology, Hua Shan Hospital of Fudan University, Shanghai, China; 4 Department of Cardiothoracic Surgery, Hua Shan Hospital of Fudan University, Shanghai, China

**Keywords:** Cell growth, Epigenetics

## Abstract

Histone lysine demethylase LSD1, also known as KDM1A, has been found to regulate multiple cancer hallmarks since it was first identified in 2004. Recently, it has emerged as a promising target for stimulating anti-tumor immunity, specifically boosting T cell activity. However, it remains unclear whether and how it remodels the tumor microenvironment to drive oncogenic processes in esophageal squamous cell carcinoma (ESCC). In this study, protein levels in ESCC tissues were evaluated by immunostaining of tissue microarrays. Cell growth was assessed by colony formation assays in vitro and subcutaneous xenograft models in vivo. High-throughput transcriptomics and spatial immune proteomics were performed using bulk RNA sequencing and digital spatial profiling techniques, respectively. Epigenetic regulation of RAD51 by methylated histone proteins was analyzed using chromatin immunoprecipitated quantitative PCR assays. Finally, our clinical data indicate that KDM1A precisely predicts the overall survival of patients with early-stage ESCC. Inhibition of KDM1A blocked the growth of ESCC cells in vitro and in vivo. Mechanistically, our transcriptomics and spatial immune proteomics data, together with rescue assays, demonstrated that KDM1A specifically removes methyl residues from the histone protein H3K9me2, a transcription repressive marker, thus reducing its enrichment at the promoter of RAD51 to epigenetically reactivate its transcription. Additionally, it significantly inhibits the expression of NF-κB signaling-dependent proinflammatory genes *IL-6* and *IL-1B* through RAD51, thus blocking the STING-associated anti-tumor immunity in stromal tumor-infiltrating lymphocytes (sTIL). Overall, our findings not only indicate that KDM1A is a promising target for ESCC patients at early stages but also provide novel mechanistic insights into its spatial regulation of STING-associated anti-tumor immunity in sTILs to drive the oncogenic processes in ESCC. The translation of these findings will ultimately guide more appropriate combinations of spatial immunotherapies with KDM1A inhibitors to improve the overall survival of specific subgroups in ESCC.

## Introduction

According to cancer statistics in China, esophageal cancer (EC) is ranked as the sixth most common cancer type and the fourth tumor-related death, with approximately 90% of patients histologically categorized as having esophageal squamous cell carcinoma (ESCC) [1]. The 3-year and 5-year overall survival (OS) rates are 65% and 54% for patients diagnosed with early-stage ESCC (Stages I and II) and 40% and 30% for those diagnosed with advanced-stage ESCC (Stages III and IV), respectively [1]. Although targeted immune strategies have been developed to achieve long-term survival for patients at advanced stages [2], the OS rates of early-stage ESCC patients are still not ideally improved because of the lack of independent prognostic markers that can be therapeutically targeted.

Lysine-specific demethylase 1 (LSD1, also known as KDM1A) is the first identified histone demethylase, with the capability to specifically remove methyl residues from H3K4me1/2, which is generally considered as an activation marker for gene transcription, and H3K9me1/2, which is linked to transcriptional repression [3], at target loci, thereby, epigenetically inhibiting or activating the expression of specific targets in a context-dependent manner [4]. Increased levels of KDM1A have been reported in multiple cancers, including ESCC [5, 6], thus igniting explorations of its inhibitors for targeted therapies [6]. Most recently, its pharmacological ablation has been shown to stimulate anti-tumor immunity by sustaining T cell invigoration, resulting in a dural response to immune checkpoint inhibitor (ICI) blockade [7, 8], making it an attractive target for enhancing immunotherapies by altering the tumor microenvironment (TME). Although high expression of KDM1A has been shown to promote malignant behaviors [9] and predict poor survivals for patients with ESCC [10], it is still unclear whether and how it remodels the TME to drive the oncogenic processes in ESCC.

Currently, cutting-edge high-throughput techniques, such as RNA sequencing (RNA-seq), T cell receptor sequencing and microarray-based spatial transcriptomics methods, at single-cell and/or spatial resolutions have been extensively applied to characterize the immune landscape in ESCC with or without chemoradiotherapies, since camrelizumab has emerged as a promising immunotherapy for ESCC patients [11–14]. Our team has also profiled the spatial immune proteins in surgically resected ESCC tissues using a multi-plex digital spatial profiling (DSP) tool and found that multiple immune proteins are both highly expressed in tumor cells (TC) and immune cells (IC) [15]. Unfortunately, it is still unclear how these spatially distributed immune proteins mediate crosstalk between TC and IC to block the oncogenic processes in ESCC.

To elucidate the role of KDM1A in remodeling the TME in ESCC, we first systematically studied its prognosis in clinicopathological and molecular stratified subgroups. Subsequently, multi-omics techniques were used to characterize the cancer hallmarks and spatial immune properties in ESCC tissues with low or high KDM1A expression. Finally, in vitro and in vivo assays were performed to elucidate the underlying mechanisms. Interestingly, our data demonstrated that KDM1A specifically removes methyl residues from the histone protein H3K9me2, thus reducing its enrichment at the promoter of RAD51 to epigenetically reactivate its transcription. In addition, it significantly inhibits the expression of NF-κB signaling dependent proinflammatory genes *IL-6* and *IL-1B* through RAD51, thus blocking the STING-associated anti-tumor immunity in stromal tumor-infiltrating lymphocytes (sTILs). Overall, our findings not only indicate that KDM1A is a promising target for ESCC patients at early stages but also provide novel mechanistic insights into its spatial regulation of STING-associated anti-tumor immunity in sTILs to drive the oncogenic processes in ESCC. The translation of these findings will ultimately guide more appropriate combinations of spatial immunotherapies with KDM1A inhibitors to improve the OS of specific subpopulations in ESCC.

## Materials and methods

### Cell culture and reagents

The human ESCC cell lines OE19, TE1, KYSE450 (K450), KYSE510 (K510), KYSE520 (K520), KYSE410 (K410), and Jurkat lymphocyte cell lines were purchased from the BeNa Culture Collection (BNCC^®^, CN). They were periodically tested mycoplasma-free using quantitative polymerase chain reaction (qPCR) assays and most recently authenticated using the short tandem repeat profiling method on a SeqStudio genetic analyzer (ThermoFisher, USA). All cells were cultured in Dulbecco’s modified Eagle’s medium supplemented with 12% fetal bovine serum (Aqlabtech, CN) and 1% penicillin-streptomycin solution (Aqlabtech, CN) at a 37 ^o^C, humidified incubator with 5% CO_2_. The ready-to-use reagents KDM1A inhibitor SP2509, ATR inhibitor (ATRi) VE-822, and PARP inhibitor (PARPi) olaparib (10 mM in DMSO) were purchased from MedChemExpress (NewJersey, USA).

### Study population, follow-up, and ethics

Archived 165 formalin fixed paraffin-embedded (FFPE) blocks from 165 patients initially diagnosed with ESCC and surgically treated at the Hua Shan Hospital of Fudan University between September 2012 and April 2020 were collected and constructed on four tissue microarrays (TMA) with one core per patient at a diameter of 2.0 mm according to our previously reported protocols [15, 16], respectively. The most recent follow-up was performed in September 2021, with survival times ranging from 2 months to 108 months. This retrospective study was approved by the Institutional Research Ethics Committee of Hua Shan Hospital of Fudan University (Protocol No. KY2022-614).

### Bulk RNA sequencing and bioinformatics

Bulk transcriptome sequencing and analysis were conducted by OE Biotech Co. Ltd (Shanghai, CN). Briefly, total RNA from TE1 and K410 stably transfected with shCtrl or shKDM1A was extracted using TRIzol reagent (Invitrogen, CA, USA), and the purity and integrity were assessed using a NanoDrop 2000 (Thermo Scientific, USA) and Agilent 2100 Bioanalyzer (Agilent Technologies, Santa Clara, CA, USA), respectively. The libraries were then established using VAHTS 150 bp Paired-end Reads Universal V6 RNA-seq Library Prep Kit according to the manufacturer’s protocol and were sequenced on an Illumina Novaseq 6000 platform. Raw reads were filtered and aligned to the human genome using HISATA2. After reads were re-assembled using StringTie2, gene structure extension, and novel transcript identification were performed using the Cuffcompare software. The read counts of each gene indicated by FPKM were calculated and obtained by HTSeq-count.

The principal component analyses (PCA) were performed using R (v.3.2.0). Differentially expressed gene (DEG) analysis was performed using DESeq2 according to the criteria of q-value < 0.05 and |log_2_[fold change (FC)]|> 0.585. Hierarchical cluster analysis of DEGs was performed using R (v.3.2.0) to visualize the individual distribution of genes in different groups. Gene Set Enrichment Analysis (GSEA) was performed using GSEA software.

### Real-time qPCR assay

According to our previously established qPCR protocol [17], the total RNA were extracted using the tissue DNA/RNA extraction kit (ONREW, CN), which was then reversed to get complementary DNA (cDNA) using the 5X All in One RT Master Mix kit (Abm, USA), followed by the amplification using the StarLighter SYBR Green qPCR Mix (Forever Star, CN) on the HONGSHI PCR instrument (SLAN-96S, CN). Taking the housekeeping gene *GAPDH* for normalization, the relative mRNA levels were calculated according to the 2^−ΔΔCT^ formula. Notably, the primers used in the study are all shown in Table S1.

### Chromatin immunoprecipitation (ChIP) assay

Chromatin from cell pellets crosslinked with formaldehyde was sonicated with Diagenode (New Jersey, USA) and precipitated with H3K9me2 or IgG antibodies (Table S2), followed by enrichment with Protein G magnetic beads (ThermoFisher, USA) and elution with ChIP buffer (Sangon, CN). The eluted DNA was digested with Proteinase K (Beyotime, CN) and purified using a Mini Elute PCR Purification Kit (Qiagen, GER) according to the manufacturer’s protocol. Finally, the DNA was amplified using SYBR real-time PCR Master Mix (Takara, JPN) and electrophoresed on an agarose gel. Using the CT values of the IgG samples as controls, the fold enrichment was evaluated using the 2^−ΔΔCT^.

### Chemotaxis assay

ESCC cells and Jurkat lymphocytes were co-cultured using BeyoGold^TM^ transwell with 8 μm pore size PET membranes (Beyotime, Shanghai, China). Briefly, 1 × 10^6^ TE1 or K410 cells stably transfected with shCtrl or shKDM1A were plated in the lower chambers in 2 ml medium, and 5 × 10^5^ Jurkat cells were seeded in the upper chambers in 1 ml medium. After 24 h, 200 μl suspension from the lower chambers was subjected to automatic cell counting and qPCR assays.

### Cell derived xenograft model

Four-week-old male BALB/c nude mice (n = 7) were ordered from Ziyuan Co., Ltd (Hangzhou, CN) and maintained at Chedun Co. Ltd (Shanghai, CN) for in vivo experiments according to the Laboratory Animal License approved by the Shanghai Science and Technology Commission [SCXK(Shanghai)2022-0001]. 3 × 10^6^ cells of K410 cells stably transfected with shCtrl or shKDM1A were mixed with Matrigel (Beyotime, Shanghai, CN) and subcutaneously injected into the right and left flank of the mice, respectively. When tumors were visible, the volumes (0.5 × length × width^2^) and weights (mg) were recorded twice per week. Sixty days after the initial injection, the mice were sacrificed according to the approved protocol when the volumes of the shCtrl group reached approximately 2000 mm^3^ (No.AD20230100). The tumors were then imaged and freshly stored at −80 ^o^C for qPCR assays or fixed with 4% paraformaldehyde for immunostaining.

### Statistics

All continuous data are presented as mean ± SD from at least three independent assays unless otherwise indicated. The significance between two groups or among three or more groups was evaluated using two-sided Student’s *t*-test and one-way ANOVA, respectively. Kaplan-Meier curve analysis was used to determine the difference in survival between the two groups. All statistical analyses were performed using GraphPad Prism version 8. The association of KDM1A with clinicopathological characteristics was analyzed using the Pearson Chi-square (χ^2^) test, and Univariate and Multivariate Cox regression analyses were used to identify independent prognostic markers using SPSS software (version 18.0). Statistical significance was categorized into four levels, p < 0.05 (*), p < 0.01 (**), p < 0.001 (***), and p < 0.0001 (****).

## Results

### High expression of KDM1A specifically predicts poor survival for ESCC patients at early stages

**Fig. 1:**
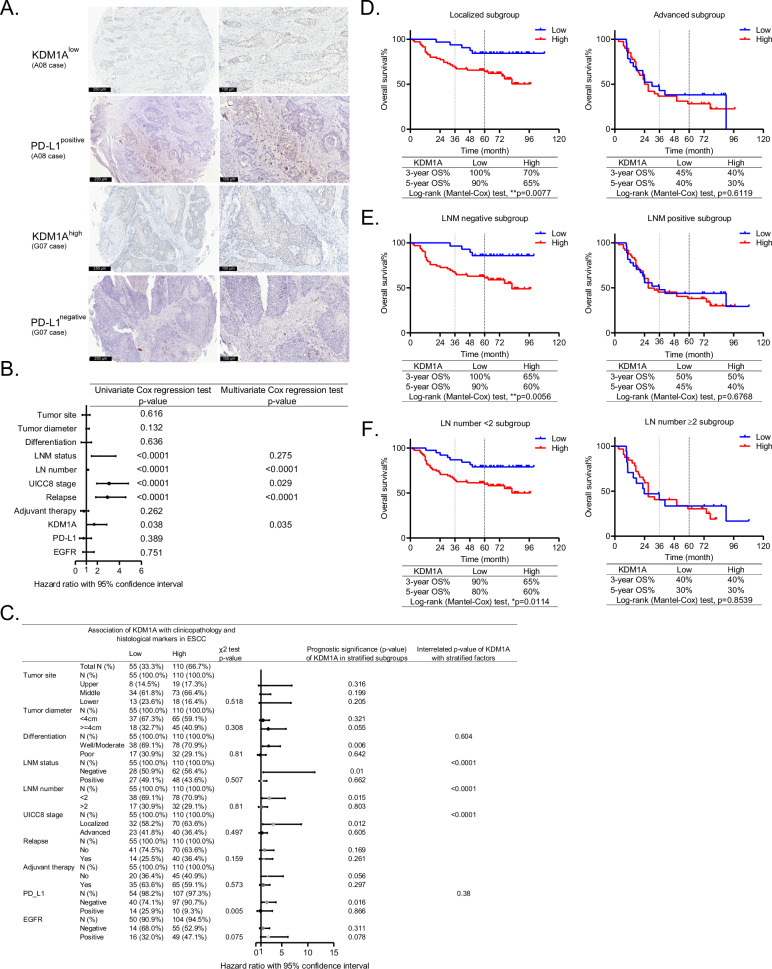
High expression of KDM1A precisely predicts poor survival for patients diagnosed with ESCC at early stages. **A** The representative immunostaining of high (KDM1A^high^) and low (KDM1A^low^) expression of KDM1A, positive (PD-L1^positive^) and negative (PD-L1^negative^) expression of PD-L1 in ESCC tissues examined by immunostaining of the consecutively sliced TMAs. Brown color represents positive staining of KDM1A (nuclear localization) or PD-L1(membrane localization). Scale bars, 250 μm (left panels) and 100 μm (right panels). **B** The forest plot of hazard ratio with 95% confidence interval (CI) showing the independent prognostic markers analyzed by the Univariate and Multivariate Cox regression method. **C** The multiplex forest plot of hazard ratio with 95% CI showing the correlation of KDM1A with clinicopathological characteristics and the differential prognosis of KDM1A in stratified subgroups. The Kaplan-Meier survival curves of ESCC patients with low/high expression of KDM1A in UICC8 stage (**D**), LNM status (**E**), and LN number (**F**) stratified subgroups.

KDM1A was first identified as a prognostic marker for ESCC in 2013 [10]. However, the precise prognosis in clinicopathological subgroups has not yet been fully documented. Most recently, its deficiency has been shown to enhance tumor immunity and T cell infiltration to elicit significant responses of checkpoint blockade-refractory mouse melanoma to anti-PD-1 therapy [8], igniting wide interest in exploring its roles in remodeling the TME. In this study, we comprehensively investigated the prognostic roles of KDM1A and PD-L1 in ESCC, and the representative immunostaining of low or high expression of KDM1A [10] and negative or positive expression of PD-L1 [15] images are shown in Fig. 1A. In line with previous reports [10], KDM1A (p = 0.035, hazard ratio (HR) = 1.714, with 95% confidence interval (CI) ranging from 1.029 to 2.854), lymph node (LN) number (p < 0.0001, HR = 1.141, with 95% CI ranging from 1.088 to 1.196), UICC8 stage (p = 0.029, HR = 3.061, with 95% CI ranging from 1.947 to 4.811) and tumor relapse (p < 0.0001, HR = 2.916, with 95% CI ranging from 1.858 to 4.577) were all identified as independent prognostic markers for patients with ESCC (Fig. 1B).

Furthermore, the precise prognosis of KDM1A in clinicopathologically stratified subgroups showed that it independently predicted unfavorable survival for patients characterized by negative LN metastasis (LNM) (Fig. 1C, p = 0.01 for the prognosis and p < 0.0001 for the interaction with KDM1A), positive LN number less than 2 (Fig. 1C, p = 0.015 for the prognosis and p < 0.0001 for the interaction with KDM1A), or localized tumors, known as UICC8 stages I and II (Fig. 1C, p = 0.012 for the prognosis and p < 0.0001 for the interaction with KDM1A). Specifically, Kaplan-Meier curves demonstrated that the 5-year OS rate of ESCC patients with high expression of KDM1A at early stages, defined as localized (Fig. 1D, left, 65% vs. 90%, p = 0.0077), LNM negative (Fig. 1E, left, 60% vs. 90%, p = 0.0056), LN number<2 (Fig. 1F, left, 60% vs. 80%, p = 0.0114), was significantly lower than that of patients with low expression of KDM1A. However, there was no difference in the 5-year OS rate for patients at late stages (p = 0.6119), defined as advanced or LNM positive (p = 0.6768) or LN number>2 (p = 0.8539) (Fig. 1D–F, right panels). Above all, our findings indicate that KDM1A specifically predicts OS in patients with early staged ESCC, which can be targeted to improve the outcomes for these subgroups.

Notably, previous studies have shown that KDM1A is positively correlated with PD-L1 in cervical cancer tissues [18] and gastric cancer specimens [19]. Besides, KDM1A ablation has been reported to stimulate anti-tumor immunity and enable the PD-1 blockade in melanoma [8]. However, our data showed that KDM1A was negatively associated with PD-L1 in ESCC (Fig. 1C, p = 0.005). Multiple clinical studies have confirmed that camrelizumab, an anti-PD-1 immune checkpoint inhibitor, has successfully improved clinical outcomes in treating patients with advanced-stage ESCC [20–22]. And here, our study showed that KDM1A is not an adverse prognostic marker for ESCC patients at advanced stages. Collectively, we thus propose that there is no need to synergize KDM1A inhibitors with ICIs to treat these subgroups in ESCC, but further preclinical and clinical trials are still required before reaching a consensus.

### Targeting KDM1A sufficiently inhibits the growth of ESCC cells in vitro and in vivo

**Fig. 2:**
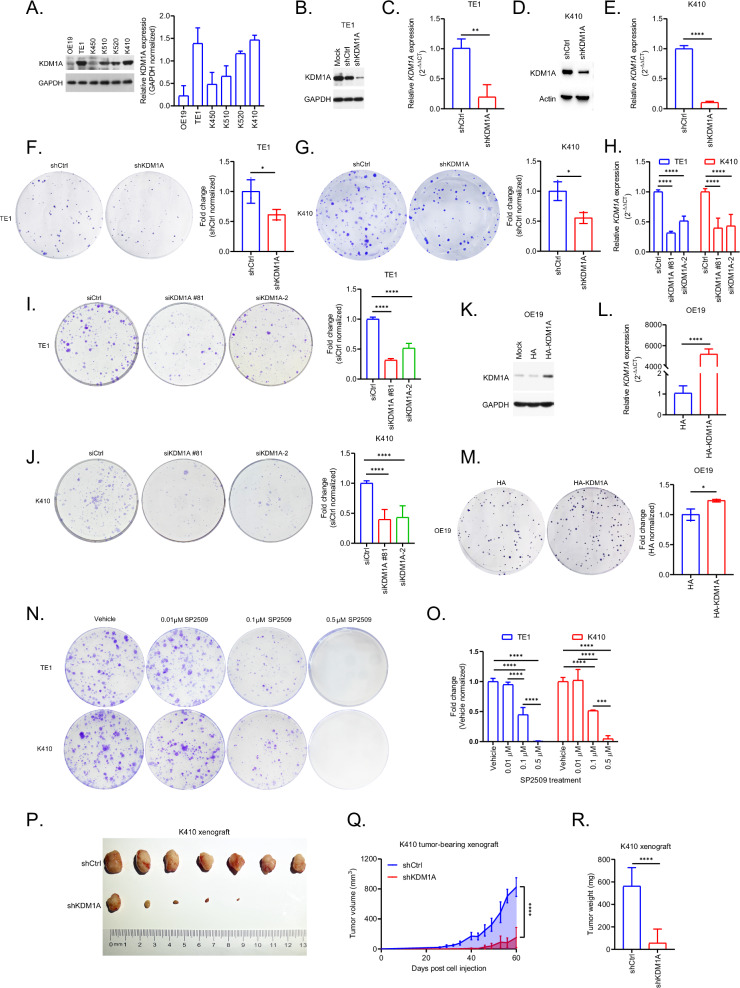
Inhibition of KDM1A sufficiently blocks the growth of ESCC cells in vitro and in vivo. **A** Representative western blotting results showing the basic levels of KDM1A in a panel of ESCC cells. Knockdown efficiency of KDM1A in TE1 and K410 cells stably transfected with shKDM1A, taking shCtrl as controls, was verified by western blotting (**B**, **D**) and qPCR assays (**C**, **E**), respectively. Representative CFA results in TE1 (**F**) and K410 (**G**) cells stably transfected with shCtrl or shKDM1A. **H** Knockdown efficiency of KDM1A in TE1 and K410 cells transfected with siKDM1A #81 or siKDM1A-2 was verified using qPCR assays, taking siCtrl as controls. Representative CFA results in TE1 (**I**) and K410 (**J**) cells transfected with siCtrl or siKDM1A #81 or siKDM1A-2. Overexpressing efficiency of KDM1A in OE19 cells transfected with HA-KDM1A, taking HA as controls, was verified by western blotting (**K**) and qPCR assays (**L**), respectively. **M** Representative CFA results in OE19 cells transfected with HA or HA-KDM1A. **N**, **O** Representative CFA results in TE1 and K410 cells continuously treated with Vehicle (0.1% DMSO) or indicated concentrations of SP2509. **P** Subcutaneous xenografts of K410 cells stably transfected with shCtrl or shKDM1A in BALB/c nude mice (n = 7). Growth curves (**Q**), shown as tumor volumes, and the tumor weight (**R**) of xenografts in (**P**). All the quantitative data are represented as mean ± SD from at least three independent assays.

To mimic clinical scenarios, we first examined the expression of KDM1A in a panel of ESCC cells. Based on the western blotting results (Fig. 2A), we divided them into KD1MA deficient (K450 and OE19) and proficient (TE1, K510, K520, and K410) models for downstream studies. Next, the knockdown efficiency of KDM1A in TE1 and K410 cells stably transfected with shCtrl or shKDM1A was validated by western blotting (Fig. 2B, D) and qPCR (Fig. 2C, E), respectively. Results from colony formation assays (CFA) showed that the deletion of KDM1A significantly inhibited the growth of TE1 (Fig. 2F, p = 0.0349) and K410 (Fig. 2G, p = 0.0129) cells. To exclude off-target effects, two additional siRNAs against KDM1A (siKDM1A #81 and siKDM1A-2), the knockdown efficiency of which was validated by qPCR (Fig. 2H, p < 0.0001 for both siKDM1As in TE1 and K410 cells), were used to confirm above CFA data. Consistently, KDM1A deficiency significantly suppressed the growth of TE1 (Fig. 2I, p < 0.0001 for both siKDM1As) and K410 cells (Fig. 2J, p < 0.0001 for both siKDM1As). Moreover, CFA results from KDM1A overexpressing OE19 cells, the efficiency of which was verified by western blotting (Fig. 2K) and qPCR (Fig. 2L, p < 0.0001), showed a significant increase in cell growth (Fig. 2M, p = 0.0142). Additionally, SP2509, which is a highly selective antagonist of KDM1A with a half-maximal enzyme activity inhibitory concentration of 13 nM in vitro [23], sufficiently inhibited the growth of both K410 and TE1 cells at low concentrations, compared to those treated with the vehicle (0.1% DMSO) (Fig. 2N–O, p < 0.0001).

In line with the above in vitro data, the growth of KDM1A deficient K410 cells was remarkably suppressed in vivo compared to those with proficient KDM1A (Fig. 2P, Q, p < 0.0001). The weights of the K410 shKDM1A xenografts were significantly lower than those of the shCtrl xenografts (Fig. 2R, p < 0.0001). Overall, our in vitro and in vivo data from multiple ESCC models sufficiently confirmed the oncogenic roles of KDM1A in ESCC, suggesting that its inhibitors are promising for improving the outcomes of patients with early staged ESCC with high KDM1A expression.

### KDM1A inhibits the expression of NF-κB-dependent inflammatory genes in ESCC

**Fig. 3:**
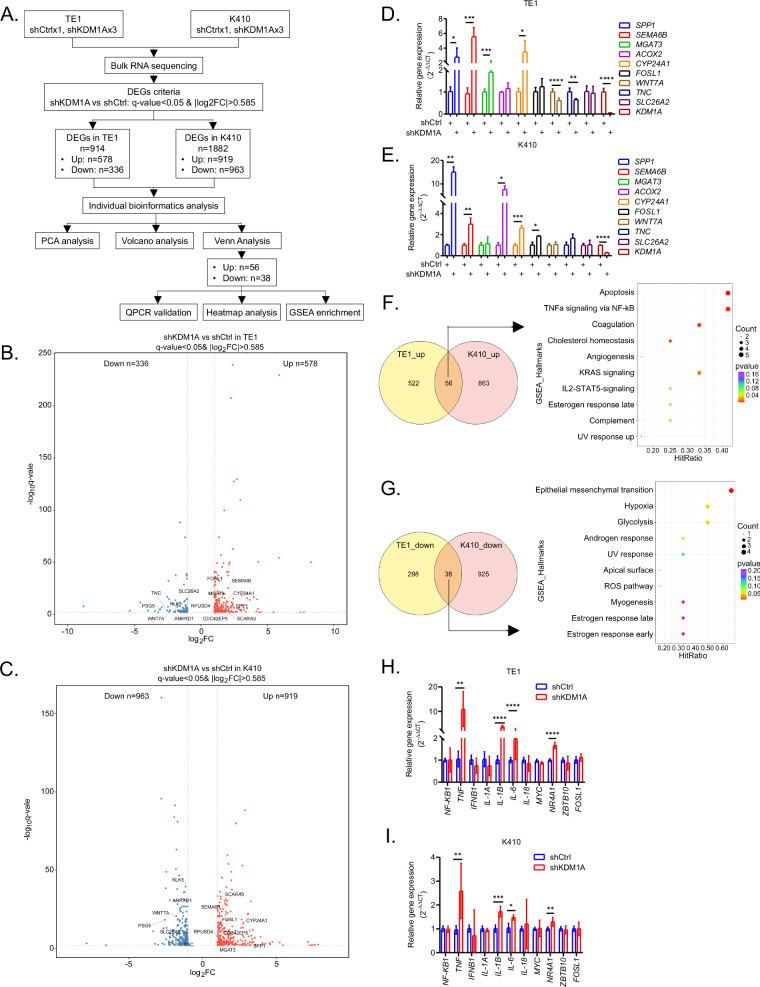
Inhibition of KDM1A significantly upregulates NF-κB mediated TNFα signaling dependent inflammatory genes in ESCC cells. **A** The flow chart showing the strategy of RNA-seq analysis in the study. The volcano plots showing the differentially expressed genes in TE1 (**B**) and K410 cells (**C**) transfected with shCtrl or shKDM1A, respectively. Validation of RNA-seq results using the qPCR assays in TE1 (**D**) and K410 (**E**) cells transfected with shCtrl or shKDM1A, respectively. The Venn diagram showing the simultaneously upregulated (**F**) or downregulated (**G**) genes in TE1 and K410 cells transfected with shKDM1A, compared to those with shCtrl, which were further subjected to GSEA enrichment analysis of top 10 cancer hallmarks with HitRatio values, respectively. The NF-κB mediated TNFα signaling dependent inflammatory genes in TE1 (**H**) and K410 (**I**) cells transfected with shCtrl or shKDM1A were tested using the qPCR assays, respectively. All the quantitative data are represented as mean ± SD from at least three independent assays.

To elucidate the potential mechanisms underlying the oncogenic roles of KDM1A in ESCC, stable TE1 and K410 cells transfected with shCtrl or shKDM1A were subjected to bulk RNA sequencing, the workflow chart is shown in Fig. 3A. Data from PCA analysis confirmed that the genes expressed in shCtrl were significantly different from those in shKDM1A in TE1 (Fig. S1A) and K410 cells (Fig. S1B). According to the DEG criteria, we found that 578 genes were upregulated and 336 genes were downregulated in KDM1A deficient TE1 cells (Fig. 3B), while 919 genes were upregulated and 963 genes were downregulated in KDM1A deleted K410 cells (Fig. 3C). To ensure RNA-seq accuracy, the top 10 significant DEGs were validated using qPCR assays, and the data showed that 70% (7 out of 10) of the genes were confirmed in TE1 cells (Fig. 3D), and 60% (6 out of 10) of the genes were validated in K410 cells (Fig. 3E).

Next, Venn analysis showed that 56 genes were simultaneously upregulated in KDM1A deficient TE1 and K410 cells, which were subjected to GSEA enrichment of cancer hallmarks and heatmap analysis. Interestingly, these upregulated genes were significantly enriched in cell apoptosis, with a HitRatio (hitgene count/total gene count) [24] of 0.417, TNFα signaling via NF-κB with a HitRatio of 0.417, coagulation with a HitRatio of 0.333, cholesterol homeostasis with a HitRatio of 0.250, angiogenesis with a HitRatio of 0.083 and KRAS signaling with a HitRatio of 0.333 (Fig. 3F). The individual visualization from the heatmaps showed that the apoptosis genes *HSPB1*, *BIK*, *TIMP3*, *PLAT*, *CLU*; NF-κB signaling genes *MYC*, *KLF9*, *NR4A1*, *ZBTB10*, *FOSL1;* coagulation genes *TIMP3*, *PLAT*, *CLU*, *ACOX2;* and KRAS signaling genes *ANO1*, *SPP1*, *PLAT*, *CA2* were highly increased in KDM1A deficient TE1 and K410 cells (Figure S1C). Additionally, 38 genes simultaneously downregulated in both cells (Fig. 3G) were significantly enriched in the epithelial mesenchymal transition (EMT) process (Fig. 3G, HitRatio=0.667). The individual visualization from the heatmaps showed that the EMT genes *TPM1*, *TNC*, *LOXL2*, and *COL5A1* were significantly downregulated (Figure S1D). Above all, we hypothesized that KDM1A probably inhibits or promotes the expression of certain genes that participate in regulating the above enriched cancer hallmarks.

Due to the significance of TNFα-NF-κB signaling in inflammation and tumor immunity [25], we next chose to focus on detecting the enriched TNFα-NF-κB signaling genes and the signaling dependent inflammatory genes. Data from qPCR assays showed that knockdown of KDM1A significantly increased the expression of the proinflammatory genes *TNF*, *IL-1B*, *IL-6*, and *NR4A1* in TE1 (Fig. 3H, p = 0.005 for *TNF*, and p < 0.0001 for *IL-6*, *IL-1B*, *NR4A1*, respectively) and K410 cells (Fig. 3I, p = 0.002 for *TNF*, p = 0.0002 for *IL-1B*, p = 0.035 for *IL-6*, and p = 0.002 for *NR4A1*, respectively), implicating that KDM1A may remodel the TME by inhibiting the expression of TNFα-NF-κB-dependent inflammatory genes, especially the *TNF*, *IL-6*, *IL-1B*, and *NR4A1* genes, in ESCC.

### KDM1A in ESCC cells is negatively correlated with STING in stromal tumor-infiltrating lymphocytes from the microenvironment

**Fig. 4:**
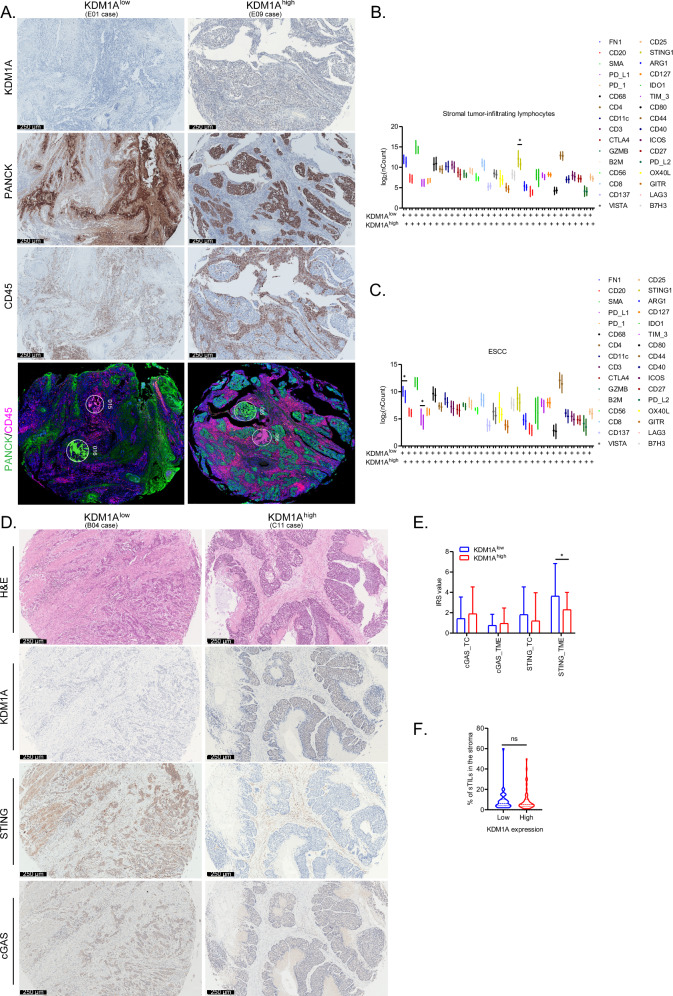
KDM1A in ESCC is spatially correlated with STING in sTILs from the TME in a negative manner. **A** The representative immunostaining of indicated proteins and the cell markers CD45 (pink) and PANCK (green) segmented ROIs from TMAs in DSP assays. Scale bar, 250 μm. The grouped plots showing the differential expression of indicated immune proteins tested by DSP assays in sTILs (**B**) and ESCC cells (**C**) from tissues with high (KDM1A^high^) or low (KDM1A^low^) expression of KDM1A. **D**, **E** Representative immunostaining results of indicated proteins in ESCC tissues with KDM1A^high^ or KDM1A^low^ (**D**). Scale bar, 250 μm. The immunoreactive scores (IRS) of indicated proteins in TC and TME of ESCC tissues (n = 83) with KDM1A^high^ or KDM1A^low^ were graphically shown (**E**). **F** The violin plots showing the differential distribution of sTILs, indicated by the percentage of positive immunostaining of CD45 per core of TMA, in ESCC tissues with low (n = 35) or high (n = 50) expression of KDM1A. All the quantitative data are represented as mean ± SD from at least three independent assays.

Taking advantage of a novel in situ DSP tool [26], we comprehensively characterized the spatial distribution of multiple immune proteins in ESCC and lymphocytes from the TME in KDM1A high and low tissues, respectively, using the cell markers PanCK indicative of ESCC (PANCK+) and CD45 of lymphocytes (CD45 + ). Representative immunostaining results of KDM1A, PANCK, CD45, and regions of interest (ROI) in PanCK+ and CD45+ regions in KDM1A low and high tissues are shown in Fig. 4A. Based on the quality criteria of the DSP tool [26], data of E02 sample were excluded (Fig. S2A). Results from PCA analysis indicated that the immune profiles in the CD45+ and PanCK+ compartments were significantly different (Fig. S2B). The individual levels of the tested factors in lymphocytes and ESCC cells are shown in Fig. S2C, D, respectively.

Differential expression analysis revealed that STING, which plays a pivotal role in mediating NF-κB signaling-dependent anti-tumor immune responses [27], was negatively related to KDM1A in sTILs (Fig. 4B, p = 0.0261), rather than in ESCC (Fig. 4C), which was further supported by immunostaining data (Fig. 4D, E, p = 0.0359). In line with above immunostaining data, the DSP data further confirmed that PD-L1 was negatively correlated with KDM1A in ESCC cells (Fig. 4C, p = 0.046), rather than in sTILs. However, the sTILs, indicated by the immunostaining of CD45, were not differentially distributed in ESCC tissues with low or high expression of KDM1A (Fig. 4F). Overall, we speculate that some inflammatory factors may mediate crosstalk between KDM1A in ESCC and STING in sTILs.

Moreover, correlation matrix analysis showed that closely correlated immune clusters were significantly different in sTILs from tissues with low and high KDM1A expression. For instance, in sTILs with low KDM1A expression (Figure S2E), PD-1, PD-L2 and CTLA4 were correlated in a cluster; CD27 was correlated with T cell activation marker CD44 [28], GZMB, GITR, B2M, ICOS, IDO1, OX40L, and CD127 were clustered with T cell markers (CD3, CD4, and CD8) [29]; LAG3 was closely correlated with the macrophage marker CD68; and CD137, TIM-3, FN1, SMA, PD-L1, ARG1, B7H3, and CD25 were clustered with the natural killer cell (NK) marker CD56 [29]. While in sTILs with high KDM1A expression (Fig. S2F), CD20, Ki-67, OX40L were correlated with monocyte markers CD56, CD68, and CD11c, PD-L1, IDO1, CD80, B2M, CD40, ICO2 were correlated with T cell markers, while CTLA4, GZMB, GITR, CD27, CD137, TIM-3, PD-L2, B7H3 were closely clustered together with dendric cells (DC) marker CD25 [29]. Taken together, we believe that KDM1A in ESCC plays a pivotal role in remodeling the immune crosstalk among T lymphocytes, NK cells, monocytes, and DCs in the TME.

### KDM1A epigenetically promotes the expression of RAD51 to drive the oncogenic processes in ESCC

KDM1A has been widely recognized to participate in homologous recombination repair (HRR) [30, 31]. Most recently, its inhibition was found to increase the sensitivity of HRR proficient ovarian cancers to PARPi by downregulating RAD51 and BRCA1/2 [32]. Consistent with these findings, the recruitment of BRCA1 (Fig. S3A, p = 0.0005 for olaparib, and p = 0.011 for VE-822) and phosphorylated RPA (pRPA) (Fig. S3B, p = 0.028 for olaparib, and p = 0.040 for VE-822) was significantly impaired in KDM1A deficient TE1 cells in response to PARPi olaparib and ATRi VE-822. In addition, cell viability was significantly reduced in KDM1A deficient TE1 cells (Fig. S3C, p < 0.0001 for VE-822, and p = 0.0005 for olaparib), whereas it was increased in KDM1A overexpressed OE19 cells (Fig. S3D, p = 0.0006 for VE-822, and p = 0.0045 for olaparib). However, KDM1A ablation did not significantly induce DNA damage in the absence of exogenous DNA damaging agents (Figure. S3A, B, untreated groups), implicating that, unlike the chemotherapeutic processes, KDM1A is not involved in the DNA damage repair process during ESCC tumorigenesis.

**Fig. 5:**
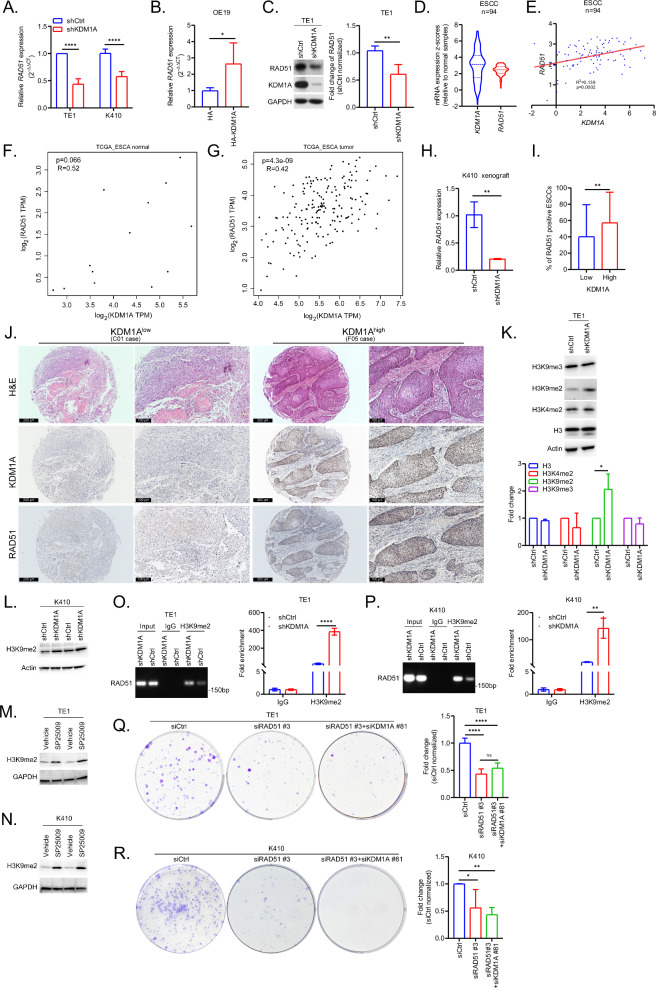
KDM1A epigenetically promotes the expression of RAD51 by demethylating H3K9me2 at its promoter to drive the oncogenic processes in ESCC. **A** The bar plots showing the significant downregulation of *RAD51*, detected by qPCR assays, in TE1 and K410 cells stably transfected with shCtrl or shKDM1A, respectively. **B** The bar plots showing the significant increase of *RAD51*, detected by qPCR assays, in OE19 cells transfected with HA or HA-KDM1A. **C** Representative western blotting results showing the downregulation of RAD51 in TE1 cells stably transfected with shCtrl or shKDM1A. The shCtrl normalized fold change data are graphically shown. **D**, **E** The violin plots showing the overexpression of *KDM1A* and *RAD51* in ESCC tissues (n = 94) from cBioportal database (**D**). The linear correlation between *KDM1A* and *RAD51* in above dataset is shown in (**E**). The correlation between *KDM1A* and *RAD51* in normal (**F**) and tumor (**G**) tissues from the TCGA_ESCA cohort. **H** The bar plots showing the significant downregulation of *RAD51*, tested by qPCR assays, in subcutaneous K410 xenografts. **I**, **J** The bar plots showing the increase of RAD51, tested by immunostaining assays of TMAs, in ESCC tissues with low (n = 55) or high (n = 110) expression of KDM1A (I), the representative immunostaining results of which are shown in (**J**). Scale bars, 200 μm and 100 μm. **K** Representative western blotting results showing the expression of indicated proteins in TE1 cells stably transfected with shCtrl or shKDM1A. The shCtrl normalized fold change data are graphically shown below. **L** The western blotting results with two repeats showing the increase of H3K9me2 in K410 cells stably transfected with shKDM1A, compared to those with shCtrl. The western blotting results with two repeats showing the increase of H3K9me2 in TE1 (**M**) and K410 (**N**) cells, treated with 0.5 μM SP2509 for 24 h, compared to those with Vehicle (0.1% DMSO). Representative agarose results of RAD51 promoter region (#1) precipitated by H3K9me2 antibody in TE1 (**O**) and K410 (**P**) cells transfected with shCtrl or shKDM1A, respectively. The fold enrichment data are graphically shown. Representative CFA results in TE1 (**Q**) and K410 (**R**) cells transfected with siCtrl or siRAD51 #3 or siRAD51 #3 and siKDM1A #81. The fold change data are graphically shown. All the quantitative data are represented as mean ± SD from at least three independent assays.

Similar to ovarian cancer [32], the expression of *RAD51* was remarkably downregulated in KDM1A deficient TE1 and K410 cells (Fig. 5A, p < 0.0001), whereas it was highly upregulated in KDM1A overexpressing OE19 cells (Fig. 5B, p = 0.0058). Moreover, data from western blotting confirmed that RAD51 expression was significantly reduced in KDM1A deficient TE1 cells (Fig. 5C, p = 0.0029). Additionally, data from cBioportal demonstrated that *KDM1A* and *RAD51* were not only highly overexpressed in ESCC tissues when compared to normal tissues (Fig. 5D), but also positively correlated with each other (Fig. 5E, p = 0.0002). More interestingly, data from TCGA_ESCA cohort suggested that the close correlation between *KDM1A* and *RAD51* was probably exclusive to ESCC tissues (Fig. 5F, G). Moreover, *RAD51* was remarkably reduced in KDM1A deleted K410 xenografts (Fig. 5H, p = 0.0039) and increased in ESCC tissues with high KDM1A expression (Fig. 5I, J, p = 0.0071). Overall, we believe that KDM1A promotes the expression of RAD51 to ensure genome stability in ESCC.

KDM1A specifically acts as an eraser of H3K4me1/2 to repress gene expression and of H3K9me1/2 to activate gene transcription [33]. Therefore, we explored the exact methylated histones modified by KDM1A to control RAD51 levels. Interestingly, data from western blotting assays showed that H3K9me2, rather than H3K4me2 or H3K9me3, was remarkably increased in KDM1A deficient TE1 cells (Fig. 5K, p = 0.0324), which was further confirmed in K410 cells (Fig. 5L). In addition, SP2509 also showed prominent induction of H3K9me2 in both TE1 (Fig. 5M) and K410 (Fig. 5N) cells, suggesting that KDM1A promotes the expression of RAD51 via H3K9me2 in ESCC. Subsequently, more direct results from ChIP-PCR assays revealed a clear increase in the interaction of RAD51 with H3K9me2 in TE1 (Fig. 5O, p < 0.0001) and K410 (Fig. 5P, p = 0.0043) cells. Further CFA data showed that deletion of KDM1A in RAD51 deficient ESCC cells did not significantly increase growth suppression in TE1 (Fig. 5Q, p < 0.0001) and K410 cells (Fig. 5R, p = 0.0278 for siRAD51 #3, p = 0.0032 for siRAD51 #3+siKDM1A #81), supporting that RAD51 is a direct downstream factor epigenetically regulated by KDM1A.

### KDM1A inhibits STING-associated anti-tumor immunity in sTILs by blocking NF-κB-dependent proinflammatory genes in ESCC

**Fig. 6:**
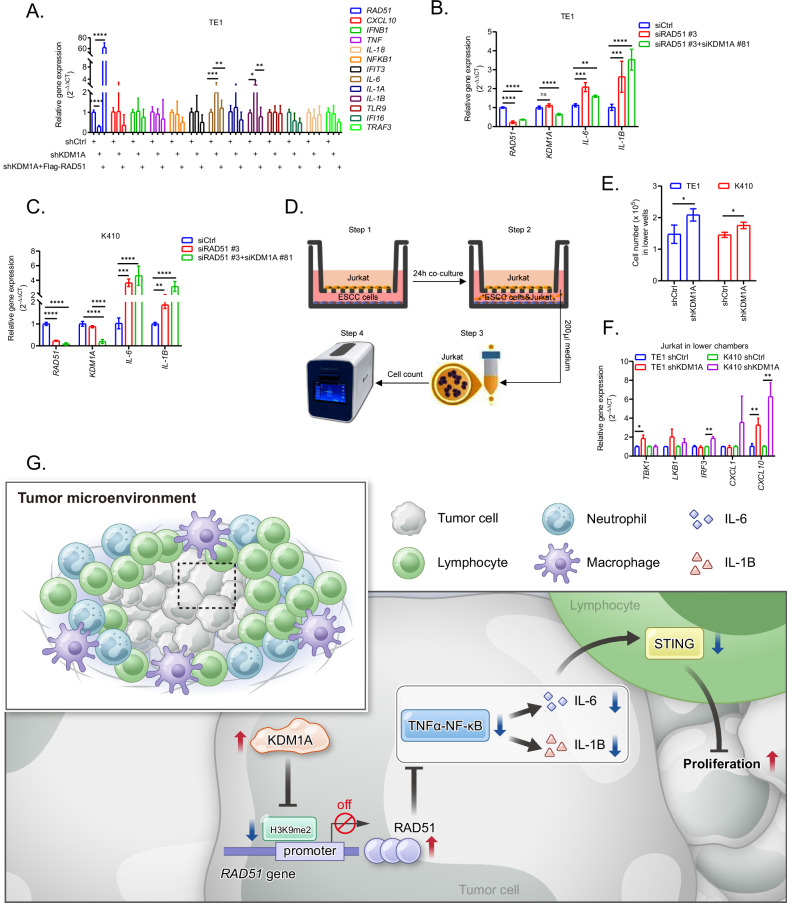
Inhibition of KDM1A significantly increases the NF-κB-dependent inflammatory genes through RAD51 to activate the STING-associated anti-tumor immunity in sTILs. **A** Relative expression of NF-κB-dependent inflammatory genes in TE1 cells transfected with shCtrl, shKDM1A, or shKDM1A+Flag-RAD51 tested by qPCR assays. Relative expression of key NF-κB-dependent inflammatory genes in TE1 (**B**) and K410 (**C**) cells transfected with siCtrl, siRAD51 #3 or siRAD51 #3+siKDM1A #81, respectively. **D** The co-culture model of ESCC cells in lower chambers with Jurkat lymphocytes in upper chambers. **E** The number of Jurkat cells in lower chambers 24 h after co-culturing with TE1 or K410 cells transfected with shCtrl or shKDM1A, respectively. **F** Relative expression of STING mediated anti-tumor immune genes in Jurkat lymphocytes from the lower chambers co-cultured with TE1 or K410 transfected with shCtrl or shKDM1A tested by qPCR assays. **G** The graphical model summarizing the mechanistic roles of KDM1A in remodeling the STING mediated anti-tumor immunity in sTILs by epigenetically upregulating RAD51 in ESCC. All the quantitative data are represented as mean ± SD from at least three independent assays.

Furthermore, data from qPCR assays showed that the increase of NF-κB-dependent proinflammatory genes *IL-6* and *IL-1B* was significantly impaired by RAD51 in KDM1A deficient cells (Fig. 6A). In line with the above CFA results in Fig. 5Q, R, deletion of KDM1A in RAD51 deficient ESCC cells did not result in obvious increases of *IL-6* and *IL-1B* in TE1 (Fig. 6B, p = 0.0001 and p = 0.0073 for *IL-6*, p = 0.0006 and p < 0.0001 for *IL-1B*, respectively) and K410 (Fig. 6C, p = 0.0001 and p < 0.0001 for *IL-6*, p = 0.006 and p < 0.0001 for *IL-1B*, respectively). Besides, the deletion of RAD51 had no effects on KDM1A expression in TE1 (Fig. 6B, p < 0.0001) and K410 (Fig. 6C, p < 0.0001), further supporting that KDM1A transcriptionally manipulates the expression of NF-κB-dependent proinflammatory genes, particularly *IL-6* and *IL-1B*, directly through RAD51.

Activation of NF-κB signaling has been reported to enhance STING signaling by altering its trafficking in macrophages [27]. And our above clinical data also showed that STING expression in sTILs was negatively associated with KDM1A expression in ESCC (Fig. 4B), thus triggering us to speculate a spatial regulation of KDM1A on the STING signaling in sTILs. Taking advantage of the co-culture model (Fig. 6D), we found that downregulation of KDM1A significantly recruited more lymphocytes to the lower chambers (Fig. 6E, p = 0.038 for TE1 and p = 0.017 for K410) and significantly increased the expression of STING-associated anti-tumor immune genes [34], particularly *CXCL10*, in Jurkat lymphocytes (Fig. 6F, p = 0.018 for *TBK1* in TE1, p = 0.002 for *IRF3* in K410, p = 0.008 and p = 0.004 for *CXCL10* in TE1 and K410, respectively), directly supporting that KDM1A remodels STING-associated anti-tumor immunity in sTILs in ESCC. Overall, our above data indicated that KDM1A deletion was unable to induce endogenous DNA damage, which can activate cGAS-STING signaling in TME [34], we thus conclude that KDM1A inhibits STING-associated anti-tumor immunity in sTILs to drive ESCC tumorigenesis by blocking NF-κB-dependent proinflammatory genes directly through RAD51, as graphically summarized in Fig. 6G.

## Discussion

A multi-omics study revealed that KDM1A is highly overexpressed in up to 51.5% of all 33 analyzed tumor types and predicts poor survival in 24.2% of them [35]. It functions as a demethylase to erase methyl residues from H3K4me1/2, which are linked to active transcription, to repress the expression of targeted genes [36], such as CoREST, HDAC, PRC2, RCOR2, NuRD, and CtBP, as well as H3K9me1/2, which is a repressive marker, to activate estrogen receptor α-dependent and androgen receptor-responsive-dependent genes [37]. Beyond histone substrates, it also demethylates non-histone substrates, such as p53, DNMT1, E2F1, STAT3, and HIF-1α in a context-dependent manner [37]. However, although it has been previously identified as an oncogenic driver and predicts unfavorable survival in patients with ESCC [10, 38], its inhibitors, such as tranylcypromine, GSK-LSD1, and SP2509, have not been clinically approved to treat these patients [39]. Here, our clinical findings suggest that KDM1A inhibitors should be specifically used to treat patients diagnosed with early-stage ESCC rather than those with advanced stages, which provides a new direction for their future clinical trials.

The clinically explored KDM1A inhibitor GSK-LSD1 has been reported to inhibit the NF-κB-dependent production of cytokines (TNFα, IL-6, IL-1) by increasing histone H3K4me2 and H3K9me2 in LPS-induced mastitis [40]. However, our data revealed that KDM1A inhibited the NF-κB-dependent proinflammatory genes *TNF*, *IL-6*, *IL-1B*, and *NR4A1* in ESCC, implicating a context-dependent mechanism. Of note, PI3K/AKT signaling, which is widely manipulated by KDM1A to inhibit autophagic cell death in TCs [41], was only enriched in TE1 cells in our study, while NF-κB signaling was enriched in both ESCC cells. Thus, we believe that the NF-κB signaling dependent expression of proinflammatory genes is more commonly regulated by KDM1A than by PI3K/AKT signaling dependent expression of autophagic genes in driving the oncogenic processes in ESCC.

Activation of NF-κB signaling has been reported to enhance STING signaling by altering STING trafficking in macrophages [27]. Therefore, if KDM1A exerts a suppressive effect on NF-κB proinflammatory signaling in ESCC, it is reasonable to think that it will block STING mediated anti-tumor immunity from the TME. Interestingly, our spatial immune proteomics study revealed that STING expression in sTILs was negatively correlated with KDM1A expression in ESCC. Notably, STING is not exclusively expressed in lymphocytes [42], it is also highly expressed in ESCC [15]. The other immune factor IDO1, which is a crucial innate immunity regulator that depletes tryptophan to suppress effector T and NK cells, as well as the differentiation and activation of regulatory T cells and myeloid-derived suppressor cells [43], has also been found to be highly expressed in both TCs and ICs. More specifically, its expression in ICs, rather than in TCs, significantly drives relapse in ESCC [15]. Therefore, the differential roles of immune proteins in TCs and ICs will ultimately promote the development of more precise spatially targeted immunotherapies in the future.

Recruitment of RAD51 to damaged DNA filaments at replication forks is the main rate-limiting step in the HRR [44]. Double-stranded DNA induced by HRR deficiency is a canonical inducer of cGAS/STING signaling-related innate immune responses [45]. Although we confirmed that deletion of KDM1A increased the sensitivity of ESCC cells to PARPi and ATRi, it was unable to induce endogenous DNA damage in the absence of DNA damaging agents. While the upregulation of NF-κB-dependent proinflammatory genes *IL-6* and *IL-1B* were significantly suppressed by RAD51 in KDM1A deleted cells, therefore, we conclude that KDM1A epigenetically regulates RAD51 expression to suppress the NF-κB-dependent proinflammatory genes *IL-6* and *IL-1B*, thus spatially blocking STING-associated anti-tumor immunity in sTILs in ESCC.

## Conclusions

In summary, the findings of this study not only indicate that KDM1A is a promising target for ESCC patients at early stages but also provide novel mechanistic insights into its spatial regulation of STING-associated anti-tumor immunity in sTILs to drive the oncogenic processes in ESCC. The translation of these findings will ultimately guide more appropriate combinations of spatial immunotherapies with KDM1A inhibitors to improve the OS of patients with early staged ESCC.

## Supplementary information

Supplementary information

Table S1

Table S2

Figure S1

Figure S2

Figure S3

qPCR original data

WB uncropped bands summary

## Data Availability

All datasets are presented in the main manuscript or supplementary files wherever possible. Bulk RNA-seq transcriptomic data were deposited in the National Genomics Data Center [46, 47] under the accession number HRA008323.
