# Supplementary material for: KDM1A epigenetically enhances RAD51 expression to suppress the STING-associated anti-tumor immunity in esophageal squamous cell carcinoma
Source: Cell Death Dis. 2024 Dec 6;15(12):882. doi: 10.1038/s41419-024-07275-4 (PMC11621790; doi:10.1038/s41419-024-07275-4)
Supplement: Supplementary file 1 — Supplementary information [file 41419_2024_7275_MOESM1_ESM.docx]

**Supplementary information**

**Supplementary methods**

**Stable cell construction**

Ready-to-use lentiviruses of shRNA targeting KDM1A (shKDM1A) and the control (shCtrl) were purchased from GeneChem (Shanghai, CN), primers of which are shown in Table S1. TE1 and K410 cells were infected with shCtrl or shKDM1A for 48h according to the manufacture’s protocol, followed by selection with 5 μg/ml puromycin (Beyotime, CN) for 10 days. The knockdown efficiency was validated using qPCR and western blotting assays.

**Plasmids construction and transfection**

Full length KDM1A (NM_015013) was amplified, primers of which are shown in Table S1, and inserted between the XhoI/BamHI sites in the GV366 vector (HA-KDM1A) (GeneChem, CN). For transfection, using the empty vector as a control (HA), 2 μg of plasmids were transfected into OE19 cells using Lipofectamine 3000 (ThermoFisher, USA) according to the manufacturer’s protocol. Overexpression efficiency was verified using both qPCR and western blotting assays.

**siRNAs and transfection**

One of the siRNAs against KDM1A (siKDM1A #81) was purchased from Sigma-Aldrich (St. Louis, MO, USA). The siRNAs against KDM1A (siKDM1A-2), RAD51 (siRAD51 #3), and the negative control (siCtrl) were synthesized by Sangon Biotech (Shanghai, CN) according to previous reports (1, 2). siRNAs (50 nM) were transfected into the indicated ESCC cells using Lipofectamine 3000 (ThermoFisher, USA) according to the manufacturer’s protocol. Knockdown efficiency was verified using qPCR assays. Sequences of all the siRNAs used in the study are shown in Table S1.

**Immunostaining assays**

Immunostaining assays were performed as previously described (3). In terms of KDM1A and RAD51 levels, the percentage (%) of nuclear positive TC of each core on TMA was defined as the protein levels. A cut-off of 75% was used to divide the patients into high and low groups, according to previous reports (4). The interpretation criteria for immune proteins cGAS, STING, and PD-L1 have been described previously (3). The TMA slides were digitally scanned using the NanoZoomer S60 scanner (Hamamatsu Photonics, JP), and representative pictures were then obtained by exporting the selected regions from the NDP.view2 software (version 2.8.24) at the indicated scale bars. The antibodies used in this assay are summarized in Table S2.

**Immunofluorescence and foci analysis**

Stable TE1 cells treated with vehicle (0.1% DMSO), 10 μM Olaparib, or 10 μM VE-822 for 4h, respectively, were fixed with 4% paraformaldehyde, followed by perforation with 0.5% Triton X-100, and incubated with primary and secondary antibodies at 37^o^C in an incubator. Finally, the cells on the coverslips were mounted with DAPI containing anti-fade solution (Beyotime, Shanghai, CN) and pictured under a 400X fluorescence microscope (Zeiss, USA). The percentage (%) of cells with foci number >10 in approximately 200-300 cells was evaluated and used for statistical analysis. The antibodies used in this assay are summarized in Table S2.

**Public datasets**

In this study, the mRNA levels of *RAD51* and *KDM1A* in ESCC tissues (n=94), indicated by z-scores relative to normal samples (log RNA Seq V2 RSEM), were downloaded from the cBioPortal database (5). The correlations between *KDM1A* and *RAD51* in TCGA_ESCA normal and tumor tissues were downloaded from GEPIA (6).

**Cell viability assays, western blotting assays, colony formation assays, and digital spatial profiling assays have been described in detail in our previously published studies (3, 7, 8).**

**References**

1. Metzger E, Wissmann M, Yin N, Muller JM, Schneider R, Peters AH, et al. LSD1 demethylates repressive histone marks to promote androgen-receptor-dependent transcription. Nature. 2005;437(7057):436-9.

2. Yang Q, Pan Q, Li C, Xu Y, Wen C, Sun F. NRAGE is involved in homologous recombination repair to resist the DNA-damaging chemotherapy and composes a ternary complex with RNF8-BARD1 to promote cell survival in squamous esophageal tumorigenesis. Cell Death Differ. 2016;23(8):1406-16.

3. Qiu C, Lin Q, Ji S, Han C, Yang Q. Expression of IDO1 in Tumor Microenvironment Significantly Predicts the Risk of Recurrence/Distant Metastasis for Patients With Esophageal Squamous Cell Carcinoma. Lab Invest. 2023;103(12):100263.

4. Yu Y, Wang B, Zhang K, Lei Z, Guo Y, Xiao H, et al. High expression of lysine-specific demethylase 1 correlates with poor prognosis of patients with esophageal squamous cell carcinoma. Biochem Biophys Res Commun. 2013;437(2):192-8.

5. Cerami E, Gao J, Dogrusoz U, Gross BE, Sumer SO, Aksoy BA, et al. The cBio cancer genomics portal: an open platform for exploring multidimensional cancer genomics data. Cancer Discov. 2012;2(5):401-4.

6. Tang Z, Li C, Kang B, Gao G, Li C, Zhang Z. GEPIA: a web server for cancer and normal gene expression profiling and interactive analyses. Nucleic Acids Res. 2017;45(W1):W98-W102.

7. Zhong M, Long, M., Han, C., Ji, S., Yang, Q. STING is significantly increased in high-grade glioma with high risk of recurrence. OncoImmunology. 2024;13(1):236658402.

8. Ji S, Fang H, Guan J, Yang Q. The immunoproteomics reveal different characteristics for the prognostic markers of intratumoral-infiltrating CD3+ T lymphocytes and Immunoscore in colorectal cancer. Lab Invest. 2024:102159.

**Supplementary figure legends**

**Figure S1. A-B.** The PCA plots showing the difference of bulk RNA-seq data in TE1 (A) and K410 (B) cells transfected with shCtrl (n=1) or shKDM1A (n=3). **C-D.** The hierarchically clustered heatmaps showing the Z-score normalized RNA-seq data of those simultaneously upregulated and downregulated genes in TE1 (C) and K410 (D) cells transfected with shCtrl or shKDM1A, respectively.

**Figure S2. A.** The ROI filtering plot showing the qualified data (blue) in DSP assays. **B.** The PCA results showing the individual DSP results from CD45 and PanCK segmented ROIs. **C-D.** The bar plots showing the individual expression of indicated immune proteins in stromal tumor-infiltrating lymphocytes (sTILs) (C) and ESCC (D) cells tested by DSP. **E-F.** The correlation matrix analysis of immune proteins in sTILs, tested by DSP assays, from ESCC tissues with low (E) or high (F) expression of KDM1A. The colored grids indicate that p<0.05. Corr_R, correlation coefficients.

**Figure S3. A-B.** Representative immunofluorescence data of BRCA1 foci (A) and pRPA (B) foci in TE1 cells stably transfected with shCtrl or shKDM1A in response to olaparib and VE-822, respectively. The percentage of positive cells (% of cells) with foci >10 is graphically shown. **C-D.** The bar plots showing the differential fold change data of cell viability, tested by CCK8 assays, in TE1 cells stably transfected with shCtrl or shKDM1A (C) and OE19 cells transfected with HA or HA-KDM1A (D) in response to VE-822 and olaparib, respectively.

**Supplementary tables**

**Table S1.** Summary of primers used in the study.

**Table S2.** Summary of antibodies used in the study.
