# Supplementary figures and images for: KDM1A epigenetically enhances RAD51 expression to suppress the STING-associated anti-tumor immunity in esophageal squamous cell carcinoma
Source: Cell Death Dis. 2024 Dec 6;15(12):882. doi: 10.1038/s41419-024-07275-4 (PMC11621790; doi:10.1038/s41419-024-07275-4)

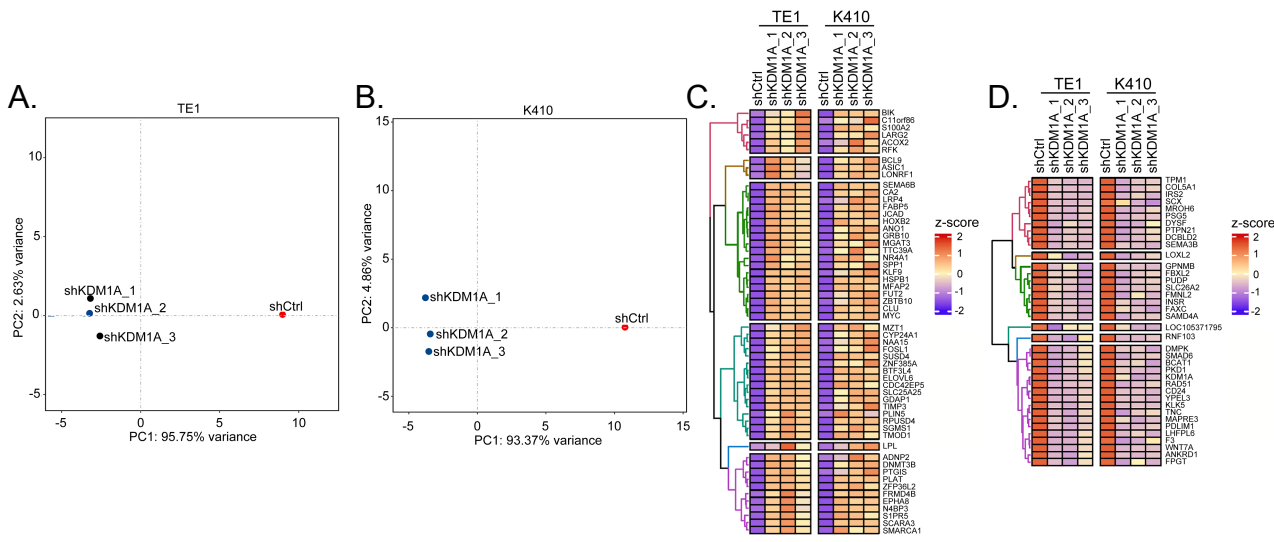

Supplement: Supplementary file 4 — Figure S1 [file 41419_2024_7275_MOESM4_ESM.pdf]

Segment ● CD45 ▲ PanCK

A.

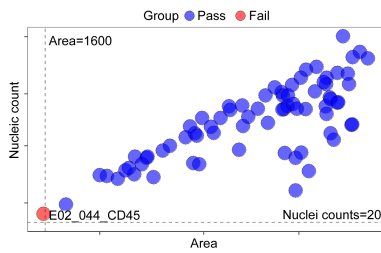

B.

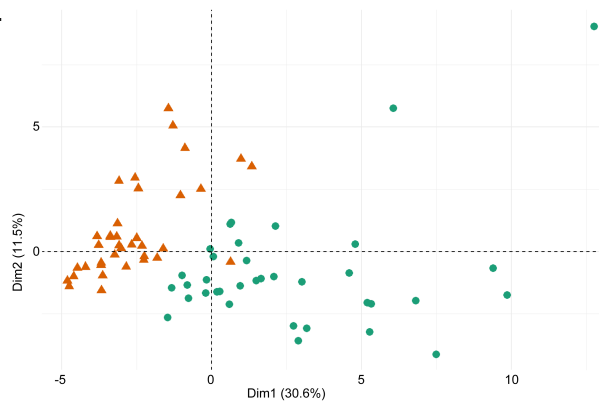

C.

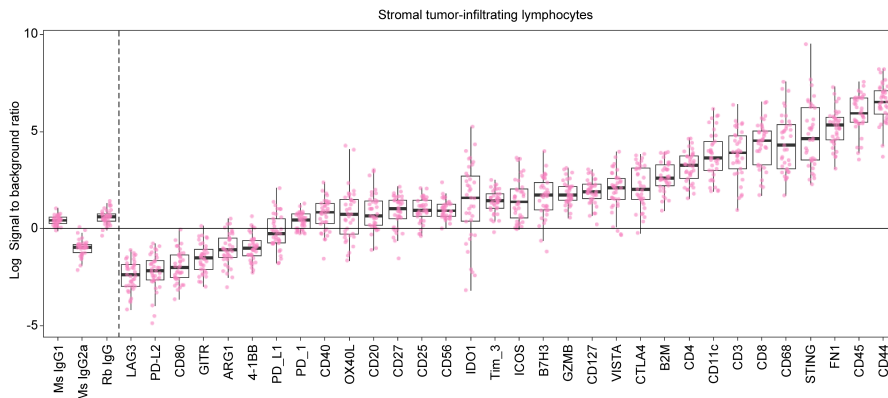

D.

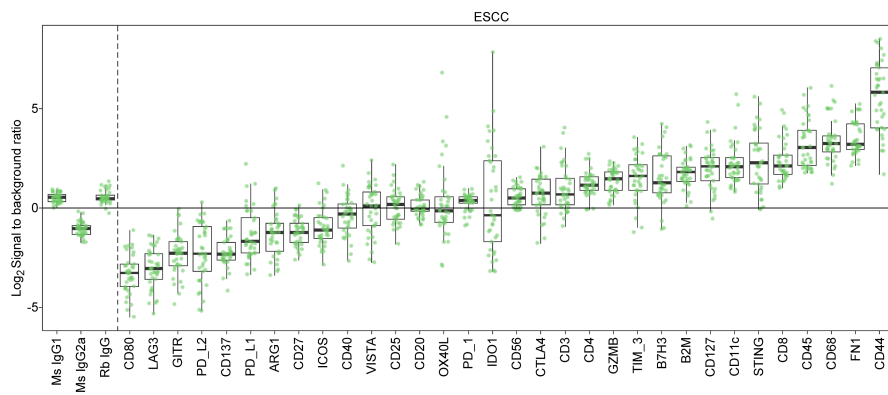

E.

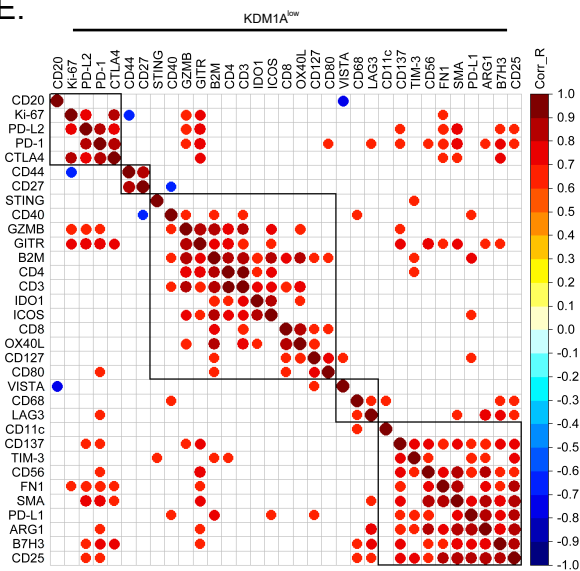

F.

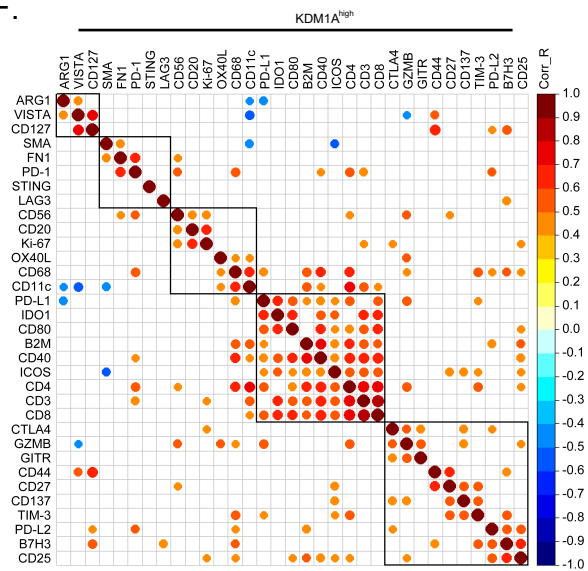

Supplement: Supplementary file 5 — Figure S2 [file 41419_2024_7275_MOESM5_ESM.pdf]

A.

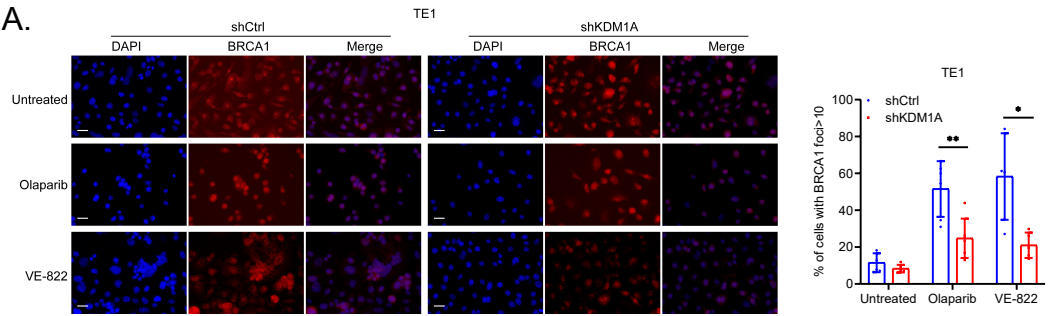

B.

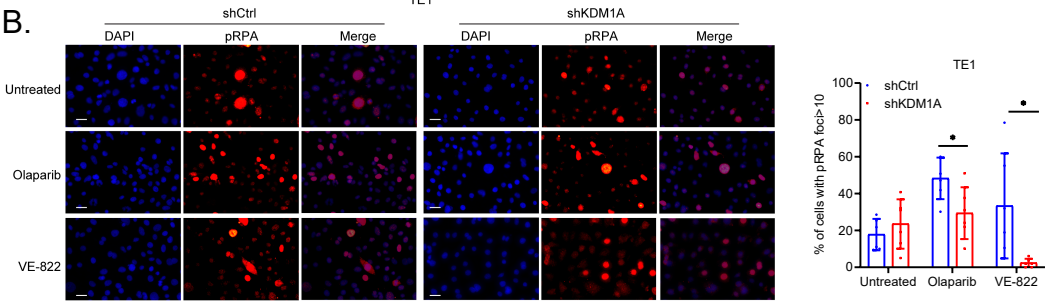

C.

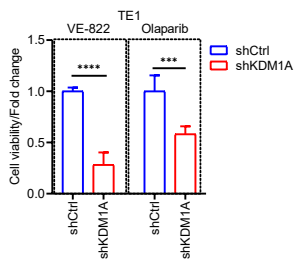

D.

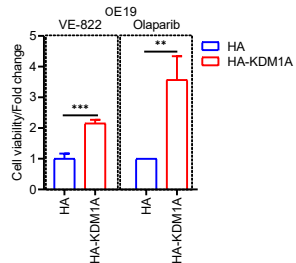

Supplement: Supplementary file 6 — Figure S3 [file 41419_2024_7275_MOESM6_ESM.pdf]

Figure 2A

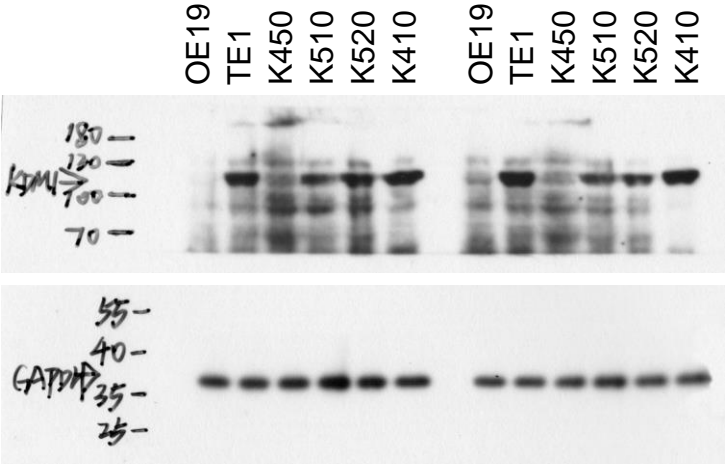

Figure 2B

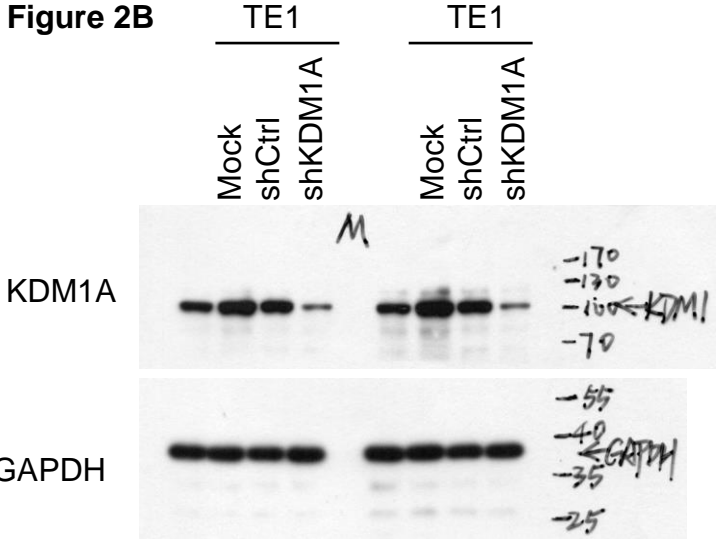

Figure 2D

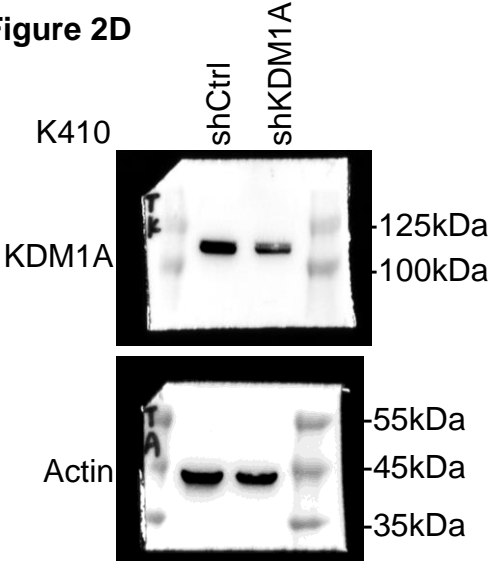

Figure 2H

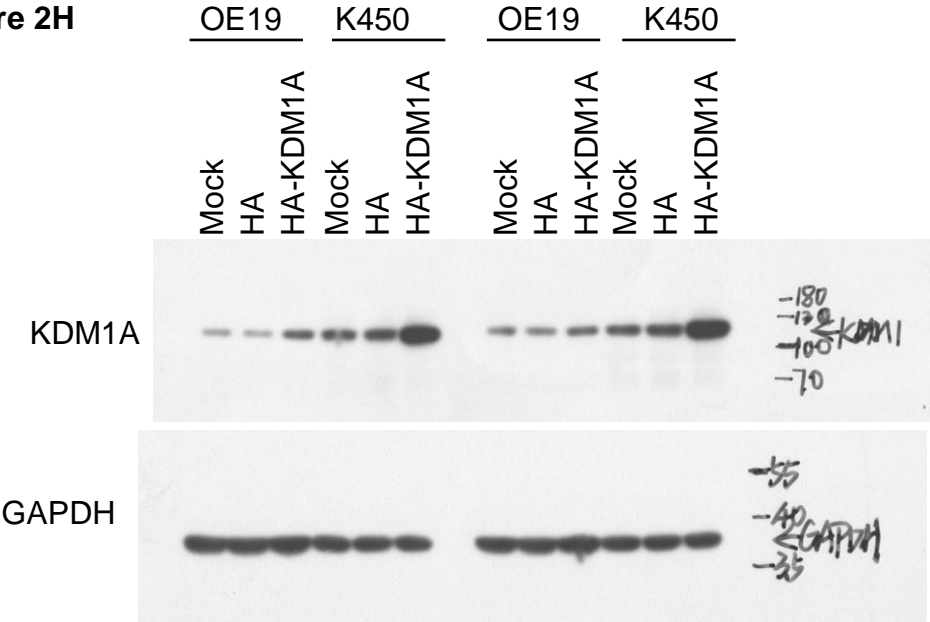

Figure 5C

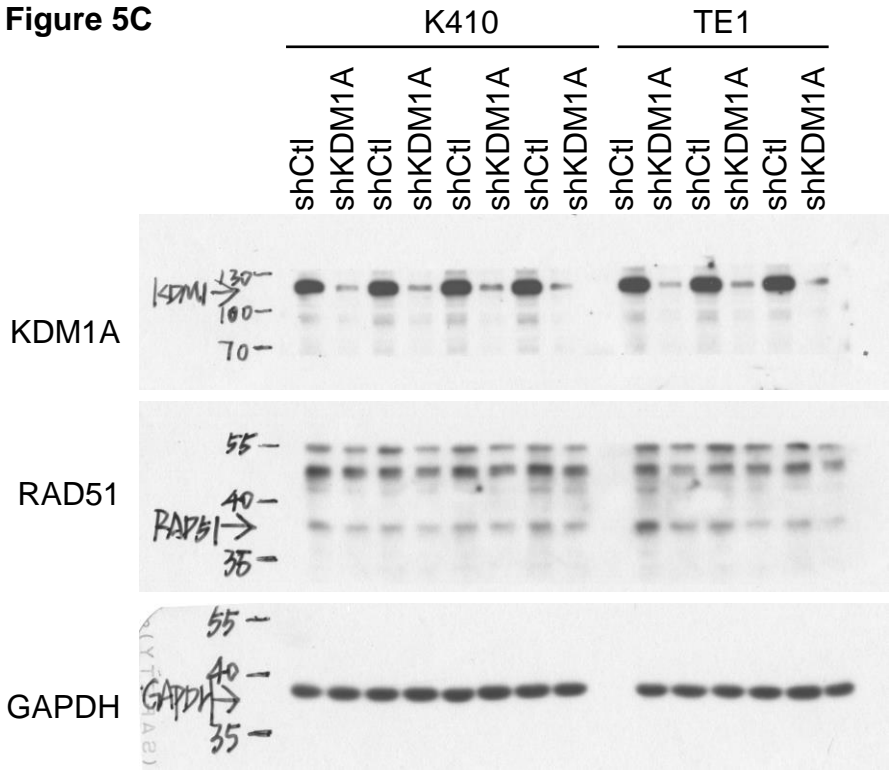

Figure 5K

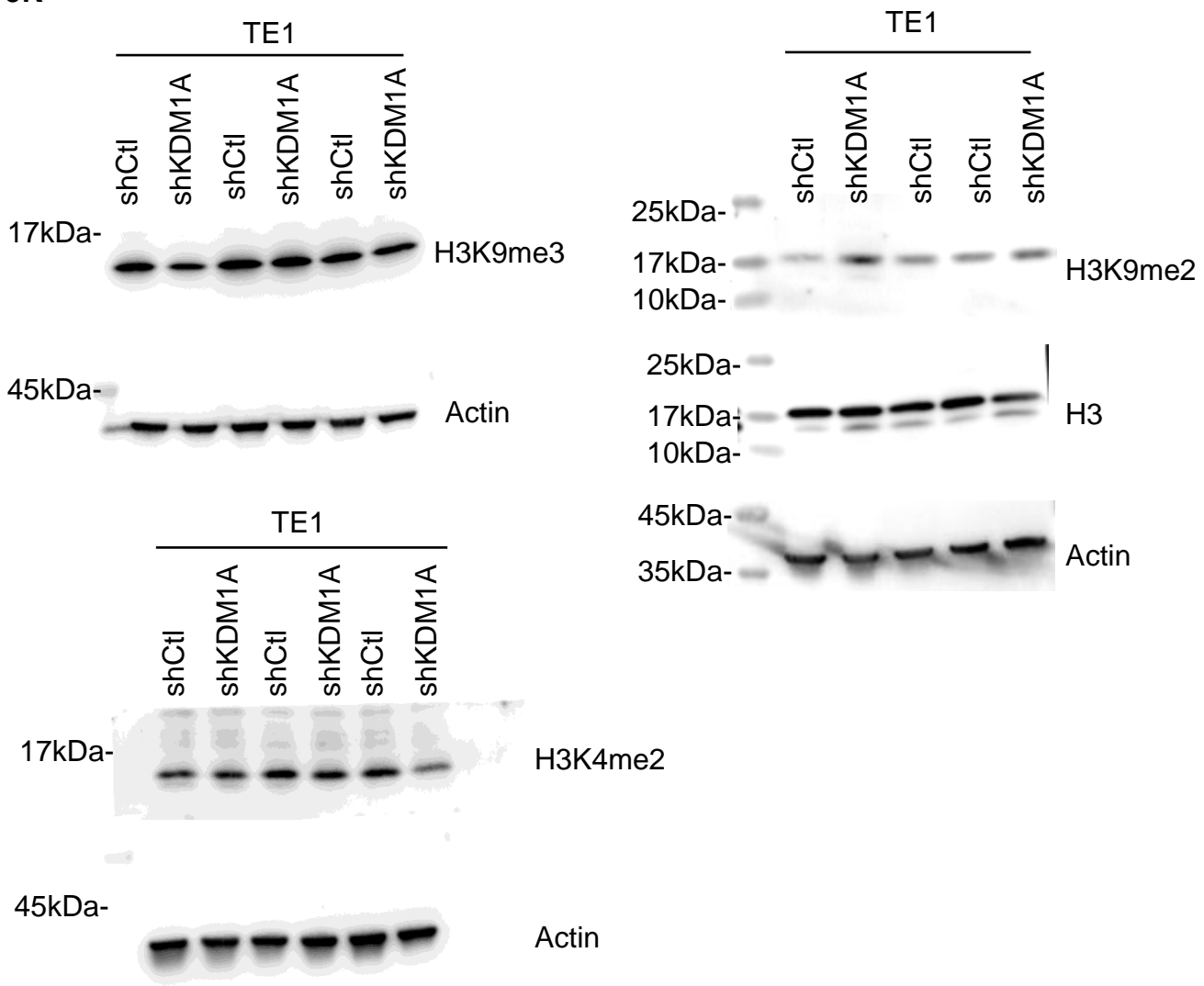

Figure 5M

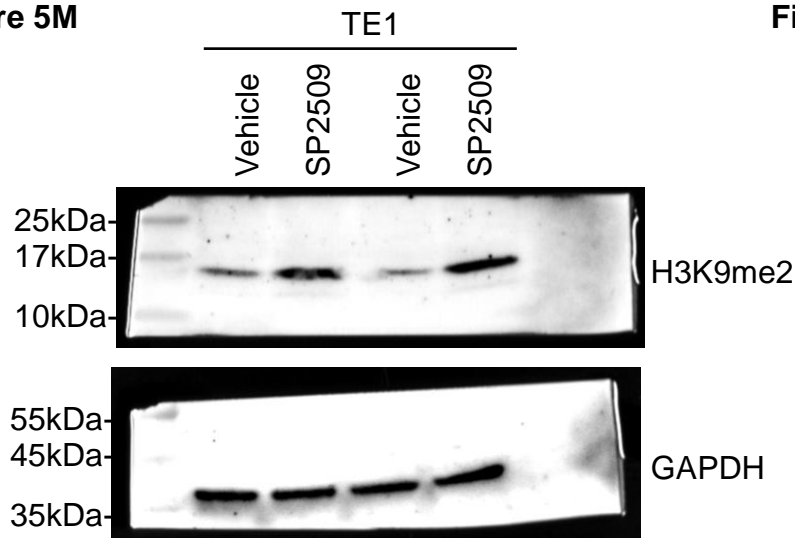

Figure 5N

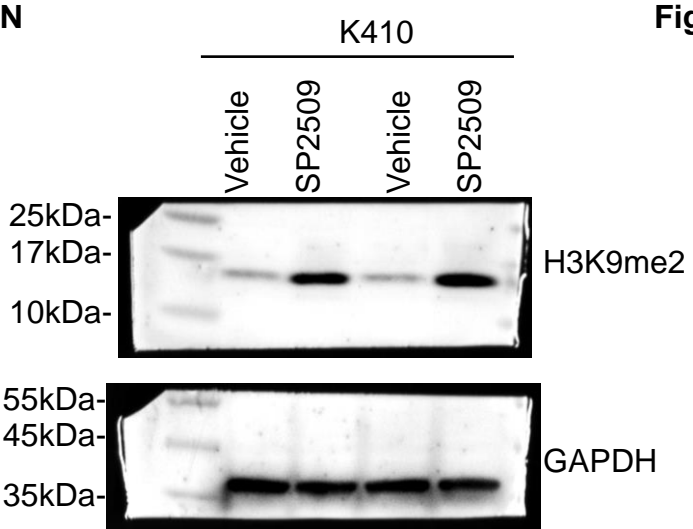

Figure 5L

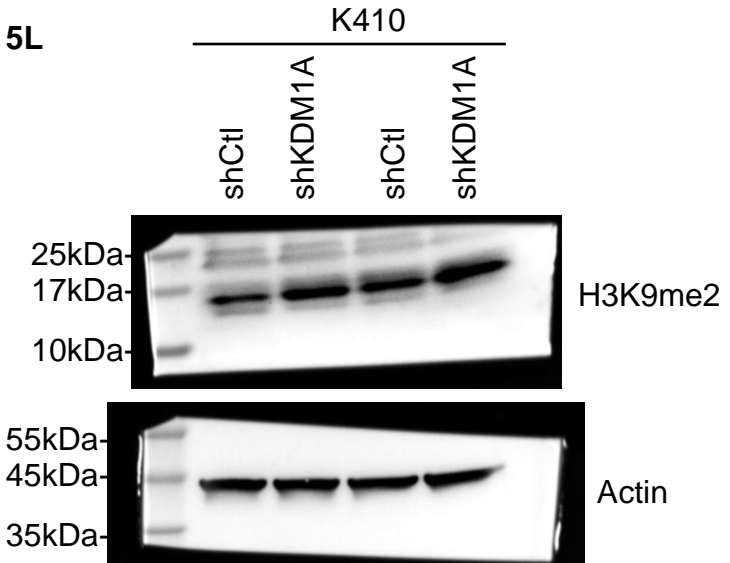

Supplement: Supplementary file 8 — WB uncropped bands summary [file 41419_2024_7275_MOESM8_ESM.pdf]
